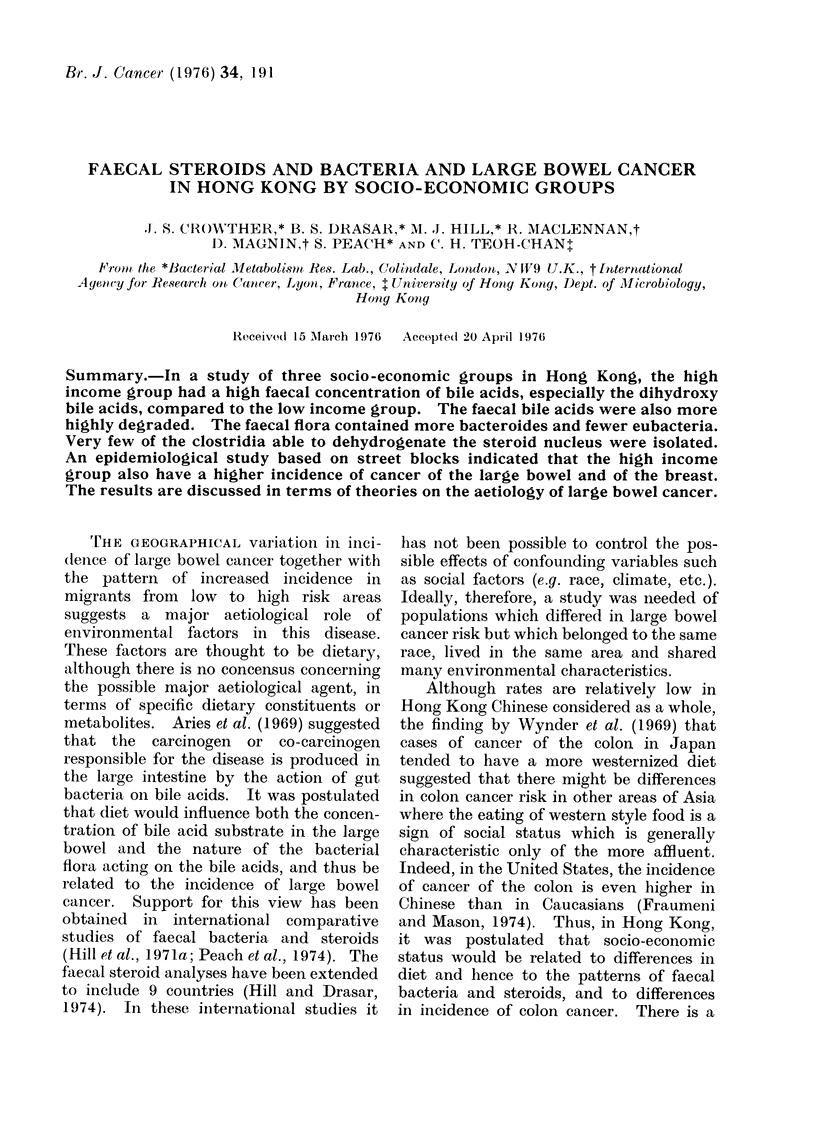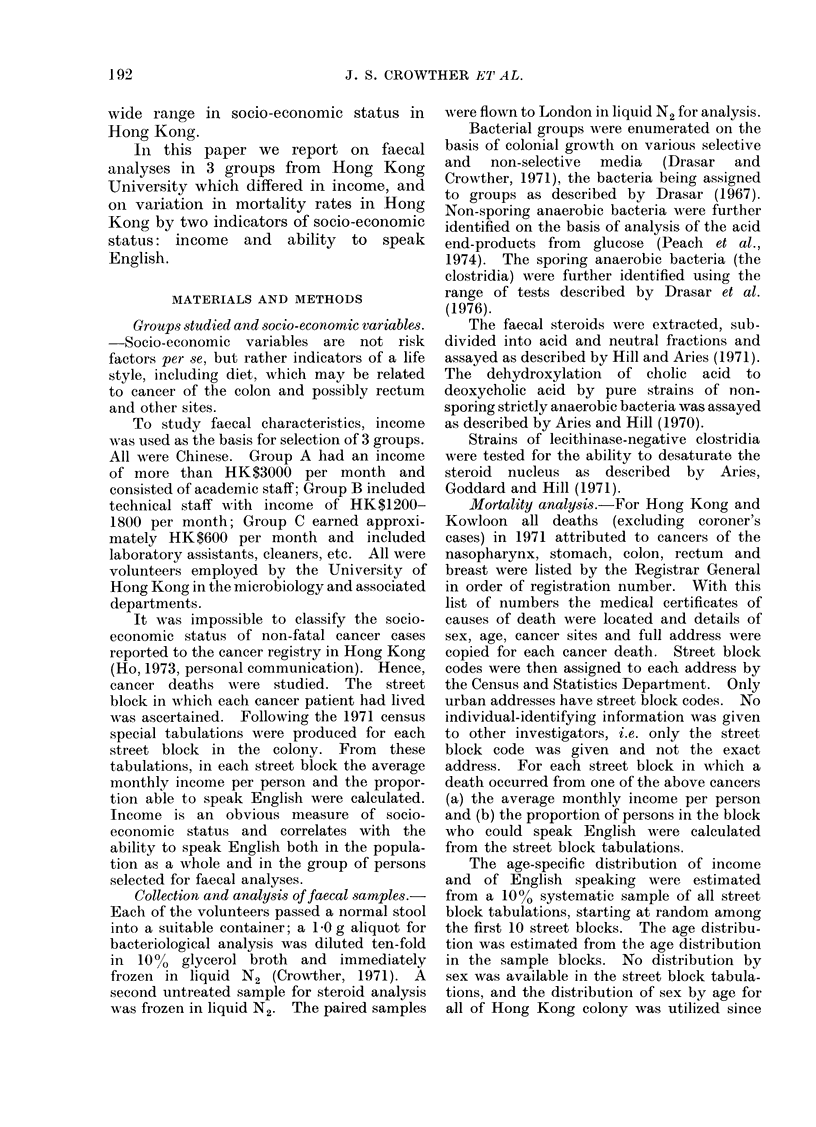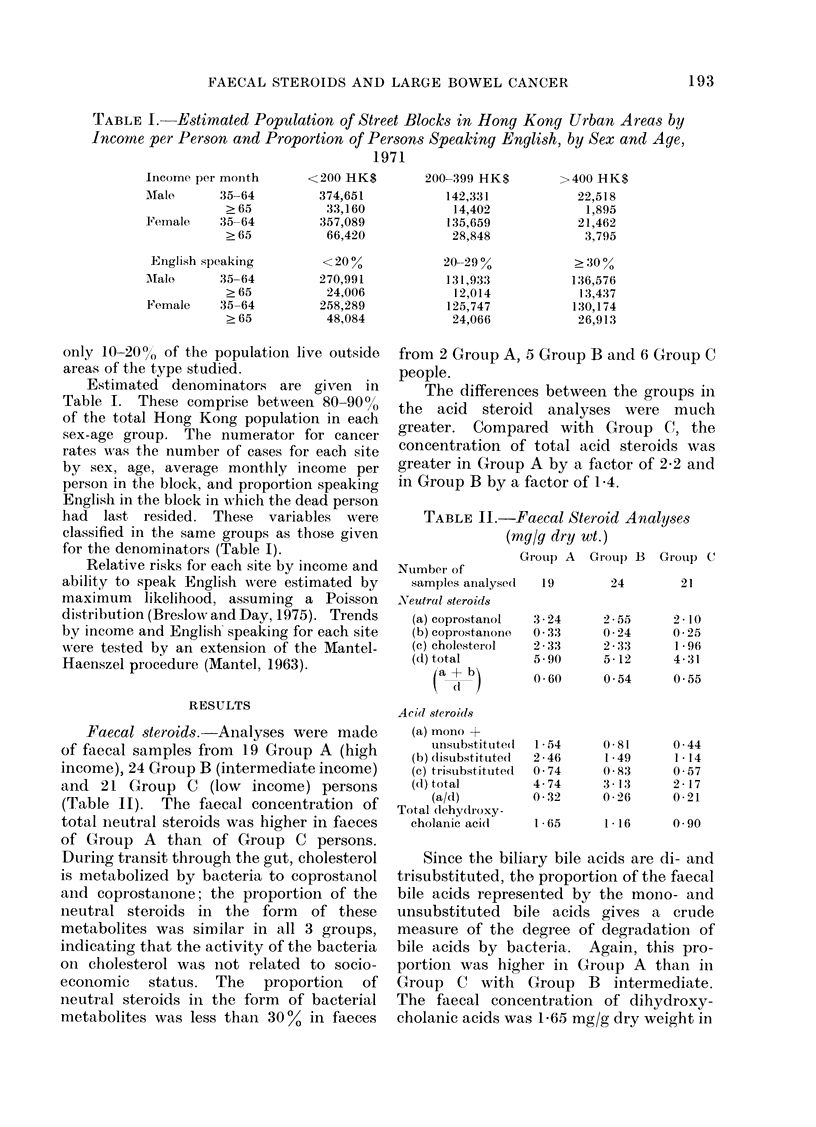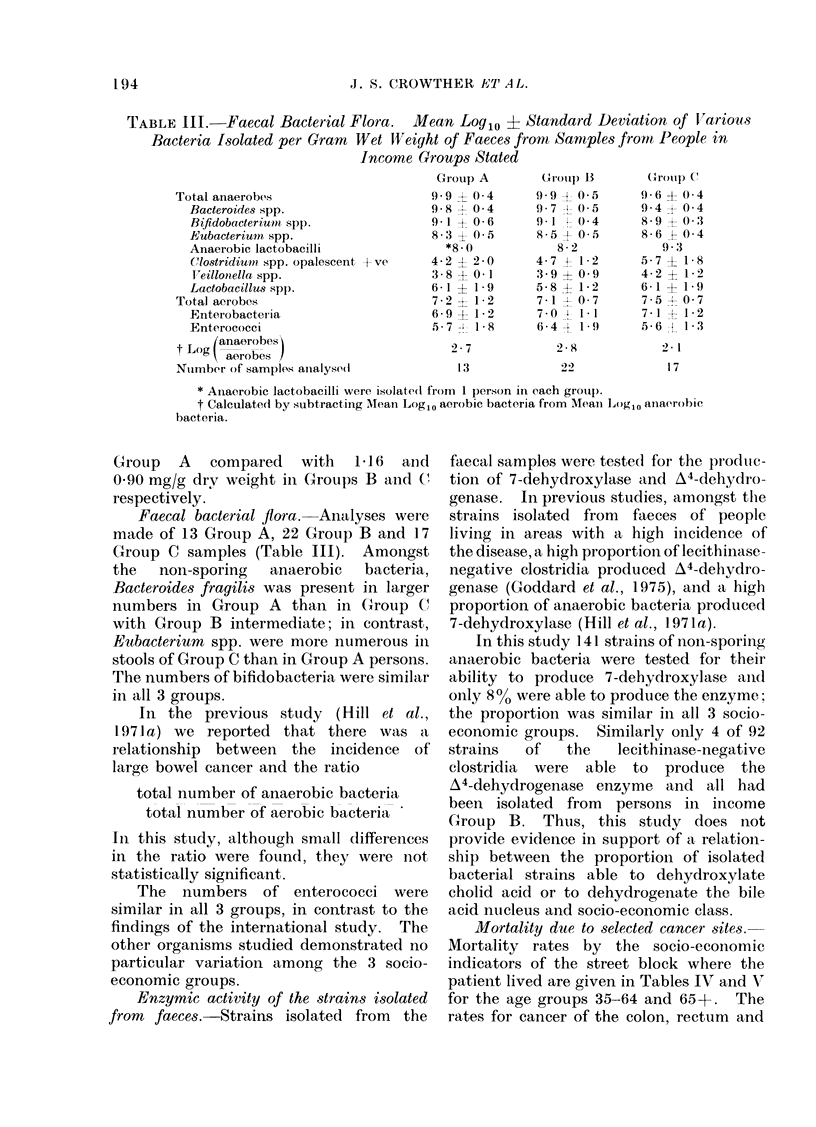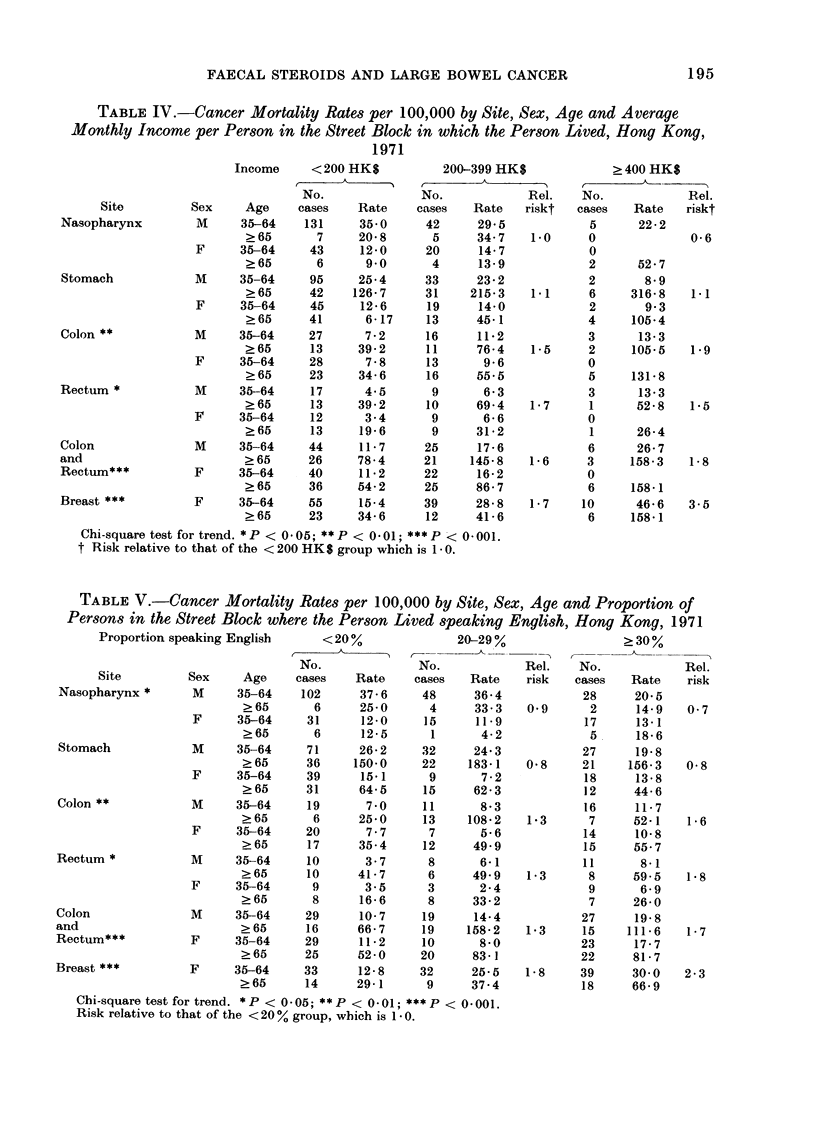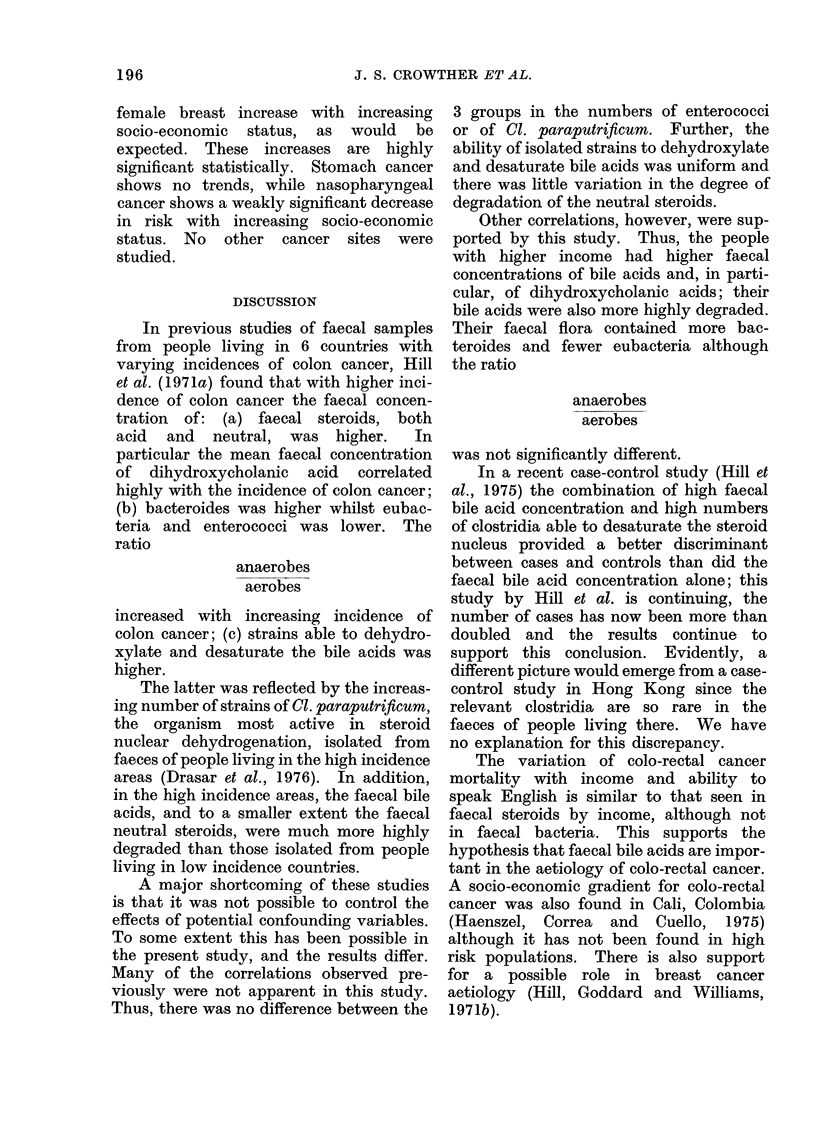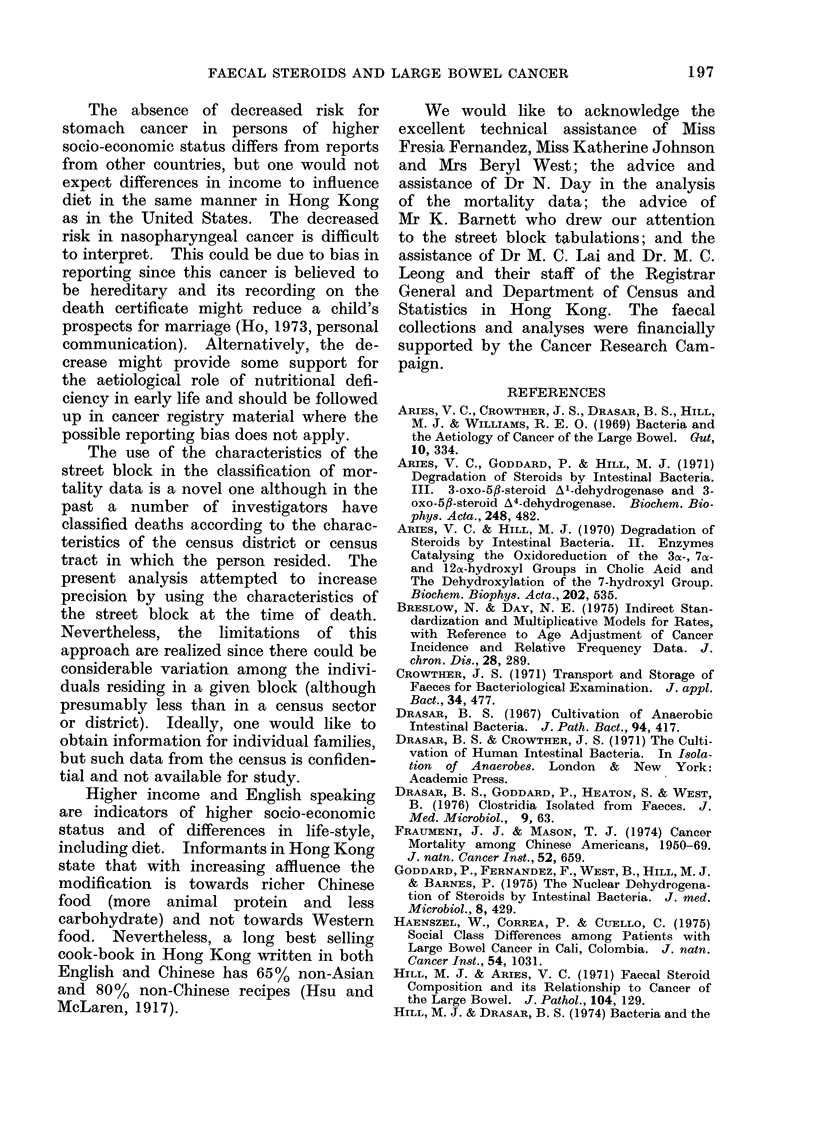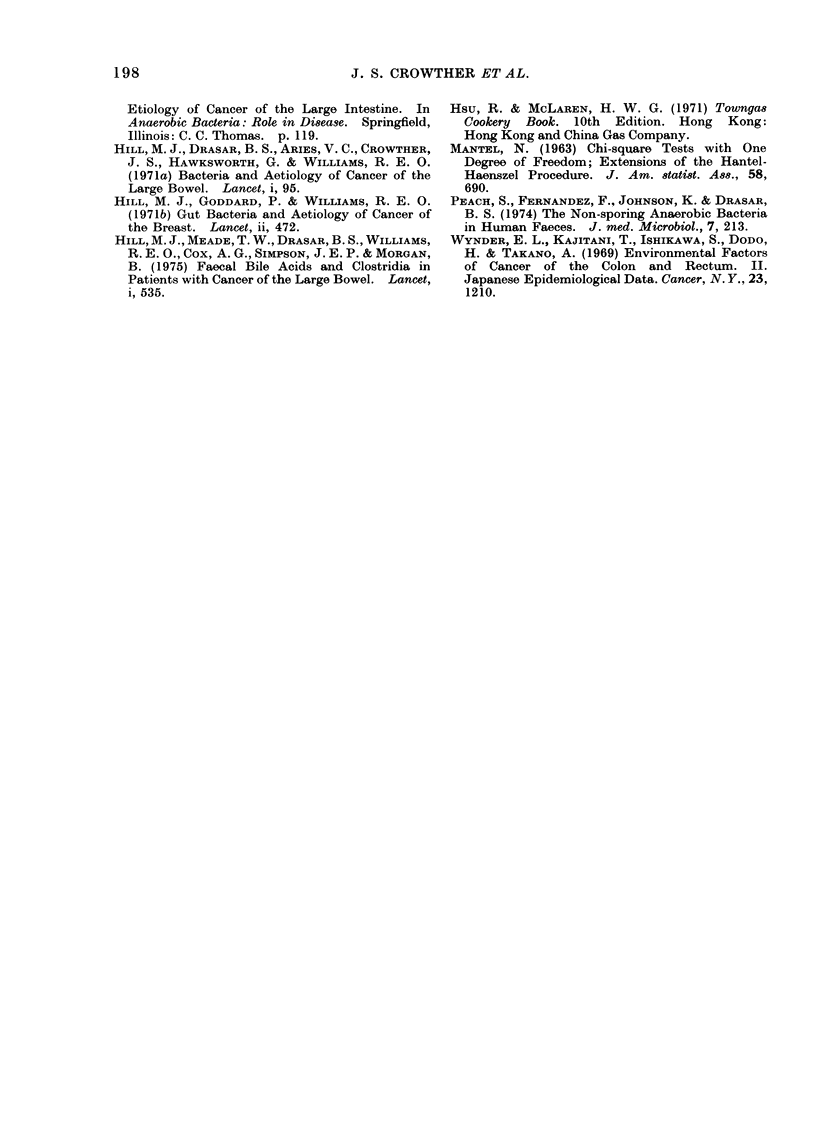# Faecal steroids and bacteria and large bowel cancer in Hong Kong by socio-economic groups.

**DOI:** 10.1038/bjc.1976.142

**Published:** 1976-08

**Authors:** J. S. Crowther, B. S. Drasar, M. J. Hill, R. Maclennan, D. Magnin, S. Peach, C. H. Teoh-chan

## Abstract

In a study of three socio-economic groups in Hong Kong, the high income group had a high faecal concentration of bile acids, especially the dihydroxy bile acids, compared to the low income group. The faecal bile acids were also more highly degraded. The faecal flora contained more bacteroides and fewer eubacteria. Very few of the clostridia able to dehydrogenate the steroid nucleus were isolated. An epidemiological study based on street blocks indicated that the high income group also have a higher incidence of cancer of the large bowel and of the breast. The results are discussed in terms of theories on the aetiology of large bowel cancer.


*Br. J. Cancer* (1976) **34**, 191

### FAECAL STEROIDS AND BACTERIA AND LARGE BOWEL CANCER IN HONG KONG BY SOCIO-ECONOMIC GROUPS

J. S. CROWTHER,* B. S. DRASAR,* M. J. HILL,* R. MACLENNAN,† D. MAGNIN,† S. PEACH* AND C. H. TEOH-CHAN‡

*From the *Bacterial Metabolism Res. Lab., Colindale, London, NW9 U.K., †International Agency for Research on Cancer, Lyon, France, ‡University of Hong Kong, Dept. of Microbiology, Hong Kong*

Received 15 March 1976 Accepted 20 April 1976

**Summary.—In a study of three socio-economic groups in Hong Kong, the high income group had a high faecal concentration of bile acids, especially the dihydroxy bile acids, compared to the low income group. The faecal bile acids were also more highly degraded. The faecal flora contained more bacteroides and fewer eubacteria. Very few of the clostridia able to dehydrogenate the steroid nucleus were isolated. An epidemiological study based on street blocks indicated that the high income group also have a higher incidence of cancer of the large bowel and of the breast. The results are discussed in terms of theories on the aetiology of large bowel cancer.**

THE GEOGRAPHICAL variation in incidence of large bowel cancer together with the pattern of increased incidence in migrants from low to high risk areas suggests a major aetiological role of environmental factors in this disease. These factors are thought to be dietary, although there is no concensus concerning the possible major aetiological agent, in terms of specific dietary constituents or metabolites. Aries *et al.* (1969) suggested that the carcinogen or co-carcinogen responsible for the disease is produced in the large intestine by the action of gut bacteria on bile acids. It was postulated that diet would influence both the concentration of bile acid substrate in the large bowel and the nature of the bacterial flora acting on the bile acids, and thus be related to the incidence of large bowel cancer. Support for this view has been obtained in international comparative studies of faecal bacteria and steroids (Hill *et al.*, 1971*a*; Peach *et al.*, 1974). The faecal steroid analyses have been extended to include 9 countries (Hill and Drasar, 1974). In these international studies it has not been possible to control the possible effects of confounding variables such as social factors (*e.g.* race, climate, etc.). Ideally, therefore, a study was needed of populations which differed in large bowel cancer risk but which belonged to the same race, lived in the same area and shared many environmental characteristics.

Although rates are relatively low in Hong Kong Chinese considered as a whole, the finding by Wynder *et al.* (1969) that cases of cancer of the colon in Japan tended to have a more westernized diet suggested that there might be differences in colon cancer risk in other areas of Asia where the eating of western style food is a sign of social status which is generally characteristic only of the more affluent. Indeed, in the United States, the incidence of cancer of the colon is even higher in Chinese than in Caucasians (Fraumeni and Mason, 1974). Thus, in Hong Kong, it was postulated that socio-economic status would be related to differences in diet and hence to the patterns of faecal bacteria and steroids, and to differences in incidence of colon cancer. There is a wide range in socio-economic status in Hong Kong.

In this paper we report on faecal analyses in 3 groups from Hong Kong University which differed in income, and on variation in mortality rates in Hong Kong by two indicators of socio-economic status: income and ability to speak English.

### MATERIALS AND METHODS

*Groups studied and socio-economic variables.*—Socio-economic variables are not risk factors *per se*, but rather indicators of a life style, including diet, which may be related to cancer of the colon and possibly rectum and other sites.

To study faecal characteristics, income was used as the basis for selection of 3 groups. All were Chinese. Group A had an income of more than HK\$3000 per month and consisted of academic staff; Group B included technical staff with income of HK\$1200–1800 per month; Group C earned approximately HK\$600 per month and included laboratory assistants, cleaners, etc. All were volunteers employed by the University of Hong Kong in the microbiology and associated departments.

It was impossible to classify the socio-economic status of non-fatal cancer cases reported to the cancer registry in Hong Kong (Ho, 1973, personal communication). Hence, cancer deaths were studied. The street block in which each cancer patient had lived was ascertained. Following the 1971 census special tabulations were produced for each street block in the colony. From these tabulations, in each street block the average monthly income per person and the proportion able to speak English were calculated. Income is an obvious measure of socio-economic status and correlates with the ability to speak English both in the population as a whole and in the group of persons selected for faecal analyses.

*Collection and analysis of faecal samples.*—Each of the volunteers passed a normal stool into a suitable container; a 1·0 g aliquot for bacteriological analysis was diluted ten-fold in 10% glycerol broth and immediately frozen in liquid $N_2$ (Crowther, 1971). A second untreated sample for steroid analysis was frozen in liquid $N_2$. The paired samples were flown to London in liquid $N_2$ for analysis.

Bacterial groups were enumerated on the basis of colonial growth on various selective and non-selective media (Drasar and Crowther, 1971), the bacteria being assigned to groups as described by Drasar (1967). Non-sporing anaerobic bacteria were further identified on the basis of analysis of the acid end-products from glucose (Peach *et al.*, 1974). The sporing anaerobic bacteria (the clostridia) were further identified using the range of tests described by Drasar *et al.* (1976).

The faecal steroids were extracted, subdivided into acid and neutral fractions and assayed as described by Hill and Aries (1971). The dehydroxylation of cholic acid to deoxycholic acid by pure strains of non-sporing strictly anaerobic bacteria was assayed as described by Aries and Hill (1970).

Strains of lecithinase-negative clostridia were tested for the ability to desaturate the steroid nucleus as described by Aries, Goddard and Hill (1971).

*Mortality analysis.*—For Hong Kong and Kowloon all deaths (excluding coroner's cases) in 1971 attributed to cancers of the nasopharynx, stomach, colon, rectum and breast were listed by the Registrar General in order of registration number. With this list of numbers the medical certificates of causes of death were located and details of sex, age, cancer sites and full address were copied for each cancer death. Street block codes were then assigned to each address by the Census and Statistics Department. Only urban addresses have street block codes. No individual-identifying information was given to other investigators, *i.e.* only the street block code was given and not the exact address. For each street block in which a death occurred from one of the above cancers (a) the average monthly income per person and (b) the proportion of persons in the block who could speak English were calculated from the street block tabulations.

The age-specific distribution of income and of English speaking were estimated from a 10% systematic sample of all street block tabulations, starting at random among the first 10 street blocks. The age distribution was estimated from the age distribution in the sample blocks. No distribution by sex was available in the street block tabulations, and the distribution of sex by age for all of Hong Kong colony was utilized since

TABLE I.—*Estimated Population of Street Blocks in Hong Kong Urban Areas by Income per Person and Proportion of Persons Speaking English, by Sex and Age, 1971*

| Income per month | | <200 HK$ | 200–399 HK$ | >400 HK$ |
|---|---|---|---|---|
| Male | 35–64 | 374,651 | 142,331 | 22,518 |
| | ≥ 65 | 33,160 | 14,402 | 1,895 |
| Female | 35–64 | 357,089 | 135,659 | 21,462 |
| | ≥ 65 | 66,420 | 28,848 | 3,795 |
| English speaking | | <20% | 20–29% | ≥30% |
| Male | 35–64 | 270,991 | 131,933 | 136,576 |
| | ≥ 65 | 24,006 | 12,014 | 13,437 |
| Female | 35–64 | 258,289 | 125,747 | 130,174 |
| | ≥ 65 | 48,084 | 24,066 | 26,913 |

only 10–20% of the population live outside areas of the type studied.

Estimated denominators are given in Table I. These comprise between 80–90% of the total Hong Kong population in each sex-age group. The numerator for cancer rates was the number of cases for each site by sex, age, average monthly income per person in the block, and proportion speaking English in the block in which the dead person had last resided. These variables were classified in the same groups as those given for the denominators (Table I).

Relative risks for each site by income and ability to speak English were estimated by maximum likelihood, assuming a Poisson distribution (Breslow and Day, 1975). Trends by income and English speaking for each site were tested by an extension of the Mantel-Haenszel procedure (Mantel, 1963).

### RESULTS

*Faecal steroids*.—Analyses were made of faecal samples from 19 Group A (high income), 24 Group B (intermediate income) and 21 Group C (low income) persons (Table II). The faecal concentration of total neutral steroids was higher in faeces of Group A than of Group C persons. During transit through the gut, cholesterol is metabolized by bacteria to coprostanol and coprostanone; the proportion of the neutral steroids in the form of these metabolites was similar in all 3 groups, indicating that the activity of the bacteria on cholesterol was not related to socio-economic status. The proportion of neutral steroids in the form of bacterial metabolites was less than 30% in faeces from 2 Group A, 5 Group B and 6 Group C people.

The differences between the groups in the acid steroid analyses were much greater. Compared with Group C, the concentration of total acid steroids was greater in Group A by a factor of 2·2 and in Group B by a factor of 1·4.

TABLE II.—*Faecal Steroid Analyses (mg/g dry wt.)*

| | Group A | Group B | Group C |
|---|---|---|---|
| Number of samples analysed | 19 | 24 | 21 |
| *Neutral steroids* | | | |
| (a) coprostanol | 3·24 | 2·55 | 2·10 |
| (b) coprostanone | 0·33 | 0·24 | 0·25 |
| (c) cholesterol | 2·33 | 2·33 | 1·96 |
| (d) total | 5·90 | 5·12 | 4·31 |
| $\left(\frac{a+b}{d}\right)$ | 0·60 | 0·54 | 0·55 |
| *Acid steroids* | | | |
| (a) mono + unsubstituted | 1·54 | 0·81 | 0·44 |
| (b) disubstituted | 2·46 | 1·49 | 1·14 |
| (c) trisubstituted | 0·74 | 0·83 | 0·57 |
| (d) total | 4·74 | 3·13 | 2·17 |
| (a/d) | 0·32 | 0·26 | 0·21 |
| Total dehydroxy-cholanic acid | 1·65 | 1·16 | 0·90 |

Since the biliary bile acids are di- and trisubstituted, the proportion of the faecal bile acids represented by the mono- and unsubstituted bile acids gives a crude measure of the degree of degradation of bile acids by bacteria. Again, this proportion was higher in Group A than in Group C with Group B intermediate. The faecal concentration of dihydroxy-cholanic acids was 1·65 mg/g dry weight in

TABLE III.—*Faecal Bacterial Flora. Mean $Log_{10}$ ± Standard Deviation of Various Bacteria Isolated per Gram Wet Weight of Faeces from Samples from People in Income Groups Stated*

| | Group A | Group B | Group C |
|---|---|---|---|
| Total anaerobes | 9·9 ± 0·4 | 9·9 ± 0·5 | 9·6 ± 0·4 |
| *Bacteroides* spp. | 9·8 ± 0·4 | 9·7 ± 0·5 | 9·4 ± 0·4 |
| *Bifidobacterium* spp. | 9·1 ± 0·6 | 9·1 ± 0·4 | 8·9 ± 0·3 |
| *Eubacterium* spp. | 8·3 ± 0·5 | 8·5 ± 0·5 | 8·6 ± 0·4 |
| Anaerobic lactobacilli | *8·0 | 8·2 | 9·3 |
| *Clostridium* spp. opalescent +ve | 4·2 ± 2·0 | 4·7 ± 1·2 | 5·7 ± 1·8 |
| *Veillonella* spp. | 3·8 ± 0·1 | 3·9 ± 0·9 | 4·2 ± 1·2 |
| *Lactobacillus* spp. | 6·1 ± 1·9 | 5·8 ± 1·2 | 6·1 ± 1·9 |
| Total aerobes | 7·2 ± 1·2 | 7·1 ± 0·7 | 7·5 ± 0·7 |
| Enterobacteria | 6·9 ± 1·2 | 7·0 ± 1·1 | 7·1 ± 1·2 |
| Enterococci | 5·7 ± 1·8 | 6·4 ± 1·9 | 5·6 ± 1·3 |
| † Log $\left(\frac{\text{anaerobes}}{\text{aerobes}}\right)$ | 2·7 | 2·8 | 2·1 |
| Number of samples analysed | 13 | 22 | 17 |

* Anaerobic lactobacilli were isolated from 1 person in each group.

† Calculated by subtracting Mean $Log_{10}$ aerobic bacteria from Mean $Log_{10}$ anaerobic bacteria.

Group A compared with 1·16 and 0·90 mg/g dry weight in Groups B and C respectively.

*Faecal bacterial flora.*—Analyses were made of 13 Group A, 22 Group B and 17 Group C samples (Table III). Amongst the non-sporing anaerobic bacteria, *Bacteroides fragilis* was present in larger numbers in Group A than in Group C with Group B intermediate; in contrast, *Eubacterium* spp. were more numerous in stools of Group C than in Group A persons. The numbers of bifidobacteria were similar in all 3 groups.

In the previous study (Hill *et al.*, 1971*a*) we reported that there was a relationship between the incidence of large bowel cancer and the ratio

$$\frac{\text{total number of anaerobic bacteria}}{\text{total number of aerobic bacteria}}.$$

In this study, although small differences in the ratio were found, they were not statistically significant.

The numbers of enterococci were similar in all 3 groups, in contrast to the findings of the international study. The other organisms studied demonstrated no particular variation among the 3 socio-economic groups.

*Enzymic activity of the strains isolated from faeces.*—Strains isolated from the faecal samples were tested for the production of 7-dehydroxylase and $\Delta^4$-dehydrogenase. In previous studies, amongst the strains isolated from faeces of people living in areas with a high incidence of the disease, a high proportion of lecithinase-negative clostridia produced $\Delta^4$-dehydrogenase (Goddard *et al.*, 1975), and a high proportion of anaerobic bacteria produced 7-dehydroxylase (Hill *et al.*, 1971*a*).

In this study 141 strains of non-sporing anaerobic bacteria were tested for their ability to produce 7-dehydroxylase and only 8% were able to produce the enzyme; the proportion was similar in all 3 socio-economic groups. Similarly only 4 of 92 strains of the lecithinase-negative clostridia were able to produce the $\Delta^4$-dehydrogenase enzyme and all had been isolated from persons in income Group B. Thus, this study does not provide evidence in support of a relationship between the proportion of isolated bacterial strains able to dehydroxylate cholid acid or to dehydrogenate the bile acid nucleus and socio-economic class.

*Mortality due to selected cancer sites.*—Mortality rates by the socio-economic indicators of the street block where the patient lived are given in Tables IV and V for the age groups 35–64 and 65+. The rates for cancer of the colon, rectum and

TABLE IV.—*Cancer Mortality Rates per* 100,000 *by Site, Sex, Age and Average Monthly Income per Person in the Street Block in which the Person Lived, Hong Kong,* 1971

| | | Income | <200 HK$ | | 200–399 HK$ | | | ≥400 HK$ | | |
|---|---|---|---|---|---|---|---|---|---|---|
| Site | Sex | Age | No. cases | Rate | No. cases | Rate | Rel. risk† | No. cases | Rate | Rel. risk† |
| Nasopharynx | M | 35–64 | 131 | 35·0 | 42 | 29·5 | | 5 | 22·2 | |
| | | ≥65 | 7 | 20·8 | 5 | 34·7 | 1·0 | 0 | | 0·6 |
| | F | 35–64 | 43 | 12·0 | 20 | 14·7 | | 0 | | |
| | | ≥65 | 6 | 9·0 | 4 | 13·9 | | 2 | 52·7 | |
| Stomach | M | 35–64 | 95 | 25·4 | 33 | 23·2 | | 2 | 8·9 | |
| | | ≥65 | 42 | 126·7 | 31 | 215·3 | 1·1 | 6 | 316·8 | 1·1 |
| | F | 35–64 | 45 | 12·6 | 19 | 14·0 | | 2 | 9·3 | |
| | | ≥65 | 41 | 6·17 | 13 | 45·1 | | 4 | 105·4 | |
| Colon ** | M | 35–64 | 27 | 7·2 | 16 | 11·2 | | 3 | 13·3 | |
| | | ≥65 | 13 | 39·2 | 11 | 76·4 | 1·5 | 2 | 105·5 | 1·9 |
| | F | 35–64 | 28 | 7·8 | 13 | 9·6 | | 0 | | |
| | | ≥65 | 23 | 34·6 | 16 | 55·5 | | 5 | 131·8 | |
| Rectum * | M | 35–64 | 17 | 4·5 | 9 | 6·3 | | 3 | 13·3 | |
| | | ≥65 | 13 | 39·2 | 10 | 69·4 | 1·7 | 1 | 52·8 | 1·5 |
| | F | 35–64 | 12 | 3·4 | 9 | 6·6 | | 0 | | |
| | | ≥65 | 13 | 19·6 | 9 | 31·2 | | 1 | 26·4 | |
| Colon | M | 35–64 | 44 | 11·7 | 25 | 17·6 | | 6 | 26·7 | |
| and | | ≥65 | 26 | 78·4 | 21 | 145·8 | 1·6 | 3 | 158·3 | 1·8 |
| Rectum*** | F | 35–64 | 40 | 11·2 | 22 | 16·2 | | 0 | | |
| | | ≥65 | 36 | 54·2 | 25 | 86·7 | | 6 | 158·1 | |
| Breast *** | F | 35–64 | 55 | 15·4 | 39 | 28·8 | 1·7 | 10 | 46·6 | 3·5 |
| | | ≥65 | 23 | 34·6 | 12 | 41·6 | | 6 | 158·1 | |

Chi-square test for trend. * $P < 0\cdot05$; ** $P < 0\cdot01$; *** $P < 0\cdot001$.
† Risk relative to that of the <200 HK$ group which is 1·0.

TABLE V.—*Cancer Mortality Rates per* 100,000 *by Site, Sex, Age and Proportion of Persons in the Street Block where the Person Lived speaking English, Hong Kong,* 1971

| | | Proportion speaking English | <20% | | 20–29% | | | ≥30% | | |
|---|---|---|---|---|---|---|---|---|---|---|
| Site | Sex | Age | No. cases | Rate | No. cases | Rate | Rel. risk | No. cases | Rate | Rel. risk |
| Nasopharynx * | M | 35–64 | 102 | 37·6 | 48 | 36·4 | | 28 | 20·5 | |
| | | ≥65 | 6 | 25·0 | 4 | 33·3 | 0·9 | 2 | 14·9 | 0·7 |
| | F | 35–64 | 31 | 12·0 | 15 | 11·9 | | 17 | 13·1 | |
| | | ≥65 | 6 | 12·5 | 1 | 4·2 | | 5 | 18·6 | |
| Stomach | M | 35–64 | 71 | 26·2 | 32 | 24·3 | | 27 | 19·8 | |
| | | ≥65 | 36 | 150·0 | 22 | 183·1 | 0·8 | 21 | 156·3 | 0·8 |
| | F | 35–64 | 39 | 15·1 | 9 | 7·2 | | 18 | 13·8 | |
| | | ≥65 | 31 | 64·5 | 15 | 62·3 | | 12 | 44·6 | |
| Colon ** | M | 35–64 | 19 | 7·0 | 11 | 8·3 | | 16 | 11·7 | |
| | | ≥65 | 6 | 25·0 | 13 | 108·2 | 1·3 | 7 | 52·1 | 1·6 |
| | F | 35–64 | 20 | 7·7 | 7 | 5·6 | | 14 | 10·8 | |
| | | ≥65 | 17 | 35·4 | 12 | 49·9 | | 15 | 55·7 | |
| Rectum * | M | 35–64 | 10 | 3·7 | 8 | 6·1 | | 11 | 8·1 | |
| | | ≥65 | 10 | 41·7 | 6 | 49·9 | 1·3 | 8 | 59·5 | 1·8 |
| | F | 35–64 | 9 | 3·5 | 3 | 2·4 | | 9 | 6·9 | |
| | | ≥65 | 8 | 16·6 | 8 | 33·2 | | 7 | 26·0 | |
| Colon | M | 35–64 | 29 | 10·7 | 19 | 14·4 | | 27 | 19·8 | |
| and | | ≥65 | 16 | 66·7 | 19 | 158·2 | 1·3 | 15 | 111·6 | 1·7 |
| Rectum*** | F | 35–64 | 29 | 11·2 | 10 | 8·0 | | 23 | 17·7 | |
| | | ≥65 | 25 | 52·0 | 20 | 83·1 | | 22 | 81·7 | |
| Breast *** | F | 35–64 | 33 | 12·8 | 32 | 25·5 | 1·8 | 39 | 30·0 | 2·3 |
| | | ≥65 | 14 | 29·1 | 9 | 37·4 | | 18 | 66·9 | |

Chi-square test for trend. * $P < 0\cdot05$; ** $P < 0\cdot01$; *** $P < 0\cdot001$.
Risk relative to that of the <20% group, which is 1·0.

female breast increase with increasing socio-economic status, as would be expected. These increases are highly significant statistically. Stomach cancer shows no trends, while nasopharyngeal cancer shows a weakly significant decrease in risk with increasing socio-economic status. No other cancer sites were studied.

### DISCUSSION

In previous studies of faecal samples from people living in 6 countries with varying incidences of colon cancer, Hill *et al.* (1971*a*) found that with higher incidence of colon cancer the faecal concentration of: (a) faecal steroids, both acid and neutral, was higher. In particular the mean faecal concentration of dihydroxycholanic acid correlated highly with the incidence of colon cancer; (b) bacteroides was higher whilst eubacteria and enterococci was lower. The ratio

$$\frac{\text{anaerobes}}{\text{aerobes}}$$

increased with increasing incidence of colon cancer; (c) strains able to dehydroxylate and desaturate the bile acids was higher.

The latter was reflected by the increasing number of strains of *Cl. paraputrificum*, the organism most active in steroid nuclear dehydrogenation, isolated from faeces of people living in the high incidence areas (Drasar *et al.*, 1976). In addition, in the high incidence areas, the faecal bile acids, and to a smaller extent the faecal neutral steroids, were much more highly degraded than those isolated from people living in low incidence countries.

A major shortcoming of these studies is that it was not possible to control the effects of potential confounding variables. To some extent this has been possible in the present study, and the results differ. Many of the correlations observed previously were not apparent in this study. Thus, there was no difference between the 3 groups in the numbers of enterococci or of *Cl. paraputrificum*. Further, the ability of isolated strains to dehydroxylate and desaturate bile acids was uniform and there was little variation in the degree of degradation of the neutral steroids.

Other correlations, however, were supported by this study. Thus, the people with higher income had higher faecal concentrations of bile acids and, in particular, of dihydroxycholanic acids; their bile acids were also more highly degraded. Their faecal flora contained more bacteroides and fewer eubacteria although the ratio

$$\frac{\text{anaerobes}}{\text{aerobes}}$$

was not significantly different.

In a recent case-control study (Hill *et al.*, 1975) the combination of high faecal bile acid concentration and high numbers of clostridia able to desaturate the steroid nucleus provided a better discriminant between cases and controls than did the faecal bile acid concentration alone; this study by Hill *et al.* is continuing, the number of cases has now been more than doubled and the results continue to support this conclusion. Evidently, a different picture would emerge from a case-control study in Hong Kong since the relevant clostridia are so rare in the faeces of people living there. We have no explanation for this discrepancy.

The variation of colo-rectal cancer mortality with income and ability to speak English is similar to that seen in faecal steroids by income, although not in faecal bacteria. This supports the hypothesis that faecal bile acids are important in the aetiology of colo-rectal cancer. A socio-economic gradient for colo-rectal cancer was also found in Cali, Colombia (Haenszel, Correa and Cuello, 1975) although it has not been found in high risk populations. There is also support for a possible role in breast cancer aetiology (Hill, Goddard and Williams, 1971*b*).

The absence of decreased risk for stomach cancer in persons of higher socio-economic status differs from reports from other countries, but one would not expect differences in income to influence diet in the same manner in Hong Kong as in the United States. The decreased risk in nasopharyngeal cancer is difficult to interpret. This could be due to bias in reporting since this cancer is believed to be hereditary and its recording on the death certificate might reduce a child's prospects for marriage (Ho, 1973, personal communication). Alternatively, the decrease might provide some support for the aetiological role of nutritional deficiency in early life and should be followed up in cancer registry material where the possible reporting bias does not apply.

The use of the characteristics of the street block in the classification of mortality data is a novel one although in the past a number of investigators have classified deaths according to the characteristics of the census district or census tract in which the person resided. The present analysis attempted to increase precision by using the characteristics of the street block at the time of death. Nevertheless, the limitations of this approach are realized since there could be considerable variation among the individuals residing in a given block (although presumably less than in a census sector or district). Ideally, one would like to obtain information for individual families, but such data from the census is confidential and not available for study.

Higher income and English speaking are indicators of higher socio-economic status and of differences in life-style, including diet. Informants in Hong Kong state that with increasing affluence the modification is towards richer Chinese food (more animal protein and less carbohydrate) and not towards Western food. Nevertheless, a long best selling cook-book in Hong Kong written in both English and Chinese has 65% non-Asian and 80% non-Chinese recipes (Hsu and McLaren, 1917).

We would like to acknowledge the excellent technical assistance of Miss Fresia Fernandez, Miss Katherine Johnson and Mrs Beryl West; the advice and assistance of Dr N. Day in the analysis of the mortality data; the advice of Mr K. Barnett who drew our attention to the street block tabulations; and the assistance of Dr M. C. Lai and Dr. M. C. Leong and their staff of the Registrar General and Department of Census and Statistics in Hong Kong. The faecal collections and analyses were financially supported by the Cancer Research Campaign.

### REFERENCES

ARIES, V. C., CROWTHER, J. S., DRASAR, B. S., HILL, M. J. & WILLIAMS, R. E. O. (1969) Bacteria and the Aetiology of Cancer of the Large Bowel. *Gut*, **10**, 334.

ARIES, V. C., GODDARD, P. & HILL, M. J. (1971) Degradation of Steroids by Intestinal Bacteria. III. 3-oxo-5$\beta$-steroid $\Delta^1$-dehydrogenase and 3-oxo-5$\beta$-steroid $\Delta^4$-dehydrogenase. *Biochem. Biophys. Acta.*, **248**, 482.

ARIES, V. C. & HILL, M. J. (1970) Degradation of Steroids by Intestinal Bacteria. II. Enzymes Catalysing the Oxidoreduction of the 3$\alpha$-, 7$\alpha$- and 12$\alpha$-hydroxyl Groups in Cholic Acid and The Dehydroxylation of the 7-hydroxyl Group. *Biochem. Biophys. Acta.*, **202**, 535.

BRESLOW, N. & DAY, N. E. (1975) Indirect Standardization and Multiplicative Models for Rates, with Reference to Age Adjustment of Cancer Incidence and Relative Frequency Data. *J. chron. Dis.*, **28**, 289.

CROWTHER, J. S. (1971) Transport and Storage of Faeces for Bacteriological Examination. *J. appl. Bact.*, **34**, 477.

DRASAR, B. S. (1967) Cultivation of Anaerobic Intestinal Bacteria. *J. Path. Bact.*, **94**, 417.

DRASAR, B. S. & CROWTHER, J. S. (1971) The Cultivation of Human Intestinal Bacteria. In *Isolation of Anaerobes*. London & New York: Academic Press.

DRASAR, B. S., GODDARD, P., HEATON, S. & WEST, B. (1976) Clostridia Isolated from Faeces. *J. Med. Microbiol.*, **9**, 63.

FRAUMENI, J. J. & MASON, T. J. (1974) Cancer Mortality among Chinese Americans, 1950–69. *J. natn. Cancer Inst.*, **52**, 659.

GODDARD, P., FERNANDEZ, F., WEST, B., HILL, M. J. & BARNES, P. (1975) The Nuclear Dehydrogenation of Steroids by Intestinal Bacteria. *J. med. Microbiol.*, **8**, 429.

HAENSZEL, W., CORREA, P. & CUELLO, C. (1975) Social Class Differences among Patients with Large Bowel Cancer in Cali, Colombia. *J. natn. Cancer Inst.*, **54**, 1031.

HILL, M. J. & ARIES, V. C. (1971) Faecal Steroid Composition and its Relationship to Cancer of the Large Bowel. *J. Pathol.*, **104**, 129.

HILL, M. J. & DRASAR, B. S. (1974) Bacteria and the

Etiology of Cancer of the Large Intestine. In *Anaerobic Bacteria: Role in Disease.* Springfield, Illinois: C. C. Thomas. p. 119.

HILL, M. J., DRASAR, B. S., ARIES, V. C., CROWTHER, J. S., HAWKSWORTH, G. & WILLIAMS, R. E. O. (1971*a*) Bacteria and Aetiology of Cancer of the Large Bowel. *Lancet*, i, 95.

HILL, M. J., GODDARD, P. & WILLIAMS, R. E. O. (1971*b*) Gut Bacteria and Aetiology of Cancer of the Breast. *Lancet*, ii, 472.

HILL, M. J., MEADE, T. W., DRASAR, B. S., WILLIAMS, R. E. O., COX, A. G., SIMPSON, J. E. P. & MORGAN, B. (1975) Faecal Bile Acids and Clostridia in Patients with Cancer of the Large Bowel. *Lancet*, i, 535.

HSU, R. & MCLAREN, H. W. G. (1971) *Towngas Cookery Book.* 10th Edition. Hong Kong: Hong Kong and China Gas Company.

MANTEL, N. (1963) Chi-square Tests with One Degree of Freedom; Extensions of the Hantel-Haenszel Procedure. *J. Am. statist. Ass.*, **58**, 690.

PEACH, S., FERNANDEZ, F., JOHNSON, K. & DRASAR, B. S. (1974) The Non-sporing Anaerobic Bacteria in Human Faeces. *J. med. Microbiol.*, **7**, 213.

WYNDER, E. L., KAJITANI, T., ISHIKAWA, S., DODO, H. & TAKANO, A. (1969) Environmental Factors of Cancer of the Colon and Rectum. II. Japanese Epidemiological Data. *Cancer, N.Y.*, **23**, 1210.